# Digital dementia training for formal caregivers: a scoping review of platforms, outcomes and evidence gaps

**DOI:** 10.1177/17449871261495844

**Published:** 2026-11-07

**Authors:** Clare Barrett, Wendy Moyle, Jonathan Foo, Marie-Claire O’Shea, Lana Mitchell

**Affiliations:** Lecturer in Nutrition and Dietetics, School of Allied Health, Sport and Social Work, Griffith University, Southport, QLD, Australia; Professor of Nursing, School of Nursing and Midwifery, Griffith University, Nathan, QLD, Australia; Lecturer, Department of Physiotherapy, Monash University, Melbourne, VIC, Australia; Senior Lecturer in Nutrition and Dietetics, School of Allied Health, Sport and Social Work, Griffith University, Southport, QLD, Australia; Senior Lecturer in Nutrition and Dietetics, School of Allied Health, Sport and Social Work, Griffith University, Southport, QLD, Australia

**Keywords:** aged, caregivers, computer-assisted instruction, continuing, dementia, education, nurses, nursing

## Abstract

**Background:**

Dementia care requires a skilled workforce, yet access to scalable and effective training for formal caregivers, including nurses, remains inconsistent. Digital education is a potential solution, but effectiveness is unclear.

**Aims:**

To examine digital dementia training programmes for formal caregivers and map their reported effects on knowledge, self-efficacy and skills.

**Methods:**

A scoping review was conducted using the Arksey and O’Malley framework and reported according to Reporting followed the Preferred Reporting Items for Systematic Reviews and Meta-Analyses extension for Scoping Reviews. Five electronic databases were searched for peer-reviewed studies evaluating fully digital dementia training interventions for formal caregivers. Methodological quality and reporting completeness were assessed using the Mixed-Methods Appraisal Tool and the Guideline for Reporting Evidence-based Educational Interventions and Teaching checklist. Findings were synthesised narratively.

**Results:**

Eighteen studies met the inclusion criteria. Digital training (online and microlearning) was associated with improvements in dementia-related knowledge and self-efficacy. Flexibility and accessibility were key advantages. However, evidence of skill development and behavioural change was limited, predominantly based on self-reported outcomes. Direct care workers were underrepresented.

**Conclusions:**

Digital dementia training offers flexible, scalable workforce education for those caring for people living with dementia. However, limited evidence on practice change constrains its application. Further research is needed to inform theory, guide implementation and support policy decisions regarding effective workforce training.

## Introduction

Dementia is a major global public health challenge, affecting 57 million people worldwide (World Health Organization, 2026). It is characterised by progressive cognitive, behavioural and functional impairments, including memory loss and disorientation, which can impact daily activities and healthcare management (World Health Organization, 2026).

Dementia is prevalent across a range of health and care settings, including residential aged care, home and community-based care, acute and subacute hospital services and specialist dementia care programmes (World Health Organization, 2022). It is a leading cause of disability and dependency among older adults globally, with care needs increasing as the condition progresses (World Health Organization, 2026). For example, in Australia, approximately 54% of residents have a diagnosis of dementia (Australian Institute of Health and Welfare, 2025), with many others receiving support through home care and community services (Australian Institute of Health and Welfare, 2025). Similar patterns are observed internationally, particularly in high-income countries (Kramarow, 2024).

Across these settings, formal caregivers play a central role in delivering day-to-day care for people living with dementia. Formal caregivers have been defined as paid workers providing care to older people in home, community or residential care settings, with varying levels of training (Li and Song, 2019). This workforce includes regulated health professionals, such as nurses, and unregulated workers, such as direct care staff, who support activities of daily living, manage behavioural and psychological symptoms, facilitate communication and coordinate care within multidisciplinary teams (Ouyang et al., 2025; Stall et al., 2019). Although roles vary across jurisdictions, these workers constitute the majority of the direct aged care workforce and are central to day-to-day care delivery (Australian Institute of Health and Welfare, 2024). Ensuring access to appropriate dementia education and training is therefore critical (World Health Organization, 2017).

Federally mandated dementia training is globally uncommon, leading to significant variability in training content, accessibility and delivery across countries and care settings (Prince et al., 2016; World Health Organization, 2017). This contributes to ongoing challenges in workforce preparation.

Formal caregivers require adequate knowledge, skills and confidence to provide high-quality dementia care (Hughes et al., 2008; World Health Organization, 2017). Accordingly, international organisations such as the World Health Organization and Alzheimer’s Disease International have emphasised workforce education as a core pillar of dementia care strategies, advocating for accessible, high-quality and scalable training options (Prince et al., 2016; World Health Organization, 2017).

Historically, face-to-face training dominated dementia education for the aged care workforce throughout the 1990s and early 2000s, although evidence of effectiveness was limited (Kuske et al., 2007). Later, Scerri and Scerri (2019) demonstrated improvements in knowledge, attitudes and confidence following face-to-face programmes, but these findings remain isolated and have not been widely replicated.

More recently, increasing demand for accessible training (Department of Health, Disability and Ageing, 2025) has driven the expansion of digital modalities, including online modules and microlearning applications. Digital training offers flexibility, self-paced learning and scalability, allowing caregivers to access educational materials alongside shift-based roles (Dementia Training Australia, n.d.; MacDonald et al., 2006; Richardson et al., 2023). Microlearning, involving brief, focused modules targeting specific competencies, has attracted attention as a particularly promising modality (De Gagne et al., 2019; McKeown, 2023a, 2023b, 2023c). These bite-sized modules are particularly suited for caregivers, as they accommodate time pressures and support repeated, manageable learning sessions (Tompkins et al., 2020). Many modules incorporate interactive components such as quizzes, case studies and simulations, enhancing engagement, comprehension and retention (De Gagne et al., 2019). As such, microlearning represents a potentially valuable modality for building dementia care capability. Alongside these potential benefits, evaluating the impact of flexible, self-paced training requires considering outcomes beyond participation and knowledge acquisition. Specifically, it is crucial to determine whether improvements in knowledge, skills and self-efficacy translate into changes in caregiving practice. In this review, behaviour refers to the application of acquired knowledge and skills in caregiving, reflected in observable changes in care delivery.

Given the importance of dementia training and the growth of digital options, a comprehensive review of existing evidence is needed to examine the effectiveness of current digital training platforms relating to this speciality.

### Aim and research questions

This review aims to identify digital training platforms for formal caregivers of people with dementia and evaluate their impact on knowledge, skills and self-efficacy. The following research questions guided the review: (1) What digital training platforms for dementia care are available globally to formal caregivers? (2) How do these platforms influence caregiver knowledge, skills and self-efficacy? and (3) Do improvements in knowledge, skills and self-efficacy translate into changes in care behaviours?

## Methods

### Design

A scoping review was conducted to clarify key concepts and terminology, given the international variation in titles, roles and job descriptions used to describe formal caregivers. This approach enabled a comprehensive overview of the existing evidence, capturing diverse study designs and methodological approaches relevant to digital dementia education. The review followed the five stages of the Arksey and O’Malley (2005) framework. The review protocol was registered prospectively with the Open Science Framework (OSF) and is available online at: https://osf.io/pzxdk/. A literature search was undertaken from December 2025 to January 2026. Reporting followed the Preferred Reporting Items for Systematic Reviews and Meta-Analyses extension for Scoping Reviews (PRISMA-ScR) guidelines (Tricco et al., 2018).

### Eligibility criteria

Eligibility criteria were developed using the Population–Concept–Context framework (Table 1). Studies were included if they: (1) involved formal caregivers of people living with dementia, including direct care workers, nurses or support staff, with data for this population reported separately; (2) evaluated a fully digital training intervention, including microlearning, intended to improve dementia-related knowledge, skills and/or self-efficacy or confidence; (3) were conducted in a relevant care setting, including residential aged care, long-term care, community care, home care, hospital, acute care or respite care; and (4) were peer-reviewed journal papers published in English. Studies were excluded if they focused only on informal caregivers, did not report formal caregiver findings separately, used multimodal interventions for which the digital component could not be isolated, were conducted outside relevant care settings, were not peer-reviewed or were not published in English. No date or study-design restrictions were applied.

**Table 1.** Eligibility criteria for studies on digital dementia training for formal caregivers.

Criterion	Inclusion criteria	Exclusion criteria
Population	Formal caregivers of people living with dementia (e.g. direct care workers, nurses, support staff), where data for this population can be isolated	Informal caregivers of people living with dementia, or studies where formal caregiver cannot be isolated
Concept	Training interventions with fully digital components (including microlearning) aimed at improving knowledge, skills and/or self-efficacy/confidence	Interventions with multiple modalities where the digital component cannot be isolated
Context	Care settings, including residential aged care, long-term care, community care, home care, hospital, acute care and respite care	Studies not conducted within relevant care settings
Study characteristics	Peer-reviewed journal papers published in English	Papers that were not peer-reviewed or were not published in English

### Search strategy

An initial exploratory search was conducted in Google Scholar to identify relevant terminology and inform the search strategy. Comprehensive searches were subsequently conducted in MEDLINE (EBSCO), CINAHL Complete (EBSCO), Scopus and ProQuest Central. These databases were selected to provide complementary coverage of the nursing, health and medical, education, social science and broader multidisciplinary literature relevant to dementia workforce training. Search strategies were developed with a health librarian. The full search strategy is provided in Supplementary Material.

PubMed, Cochrane Library and Web of Science were excluded due to content overlap. Given the limited volume of relevant research identified during the exploratory search, no date restrictions or study-design restrictions were applied to maximise the breadth of evidence captured. Keywords, synonyms and relevant MeSH terms were searched in the titles and abstracts (Table 2).

**Table 2.** Search terms.

Term	Title/abstract	MeSH/Exact subject heading/MAINSUBJECT/keyword
Dementia	Dementia	Dementia
	Alzheimer*	Dementia nursing
	BPSD	Alzheimer’s disease
	Behavioural and psychological	Neurocognitive disorders
	Symptoms of dementia	Geriatric psychiatry
Training	Education	Education
	Training	In-service training
		Training
		Continuing education
		Clinical competence education
		Continuing – organisation and administration
		Geriatric nursing education
		Nurses’ aides – education
		Job training
		Educational programmes
		Competency-based learning
Digital education	Online	Education, distance
	Digital	Internet – organisation and administration
	Microlearning	Online instruction
	Microlearning	Application
	Microlearning	Distance learning
	Microeducation	Internet
	Bite-sized learning	Distance education
	Spaced learning	Online learning
	Spaced education	Learning online
	Spaced repetition	Online education
	Distributed learning	Education online
	Just-in-time learning	
	JIT learning	
	Nano learning	
	Micro-content	
	Micro-content	
	Micro-course	
	Micro-course	
	Learning nuggets	
	Mobile microlearning	
	App-based learning	
Formal caregivers	Caregiver*	Caregivers
		Health personnel
	Carer*	Occupational groups
	Nurs*	Caregiver
	Worker*	Nurse
	Staff	Personnel
	Assist*	Worker
	Personnel	Nurses’ aides – psychology
	Aide	Workforce
	Employee	Care giver
	PCW	Care aide
	AIN	Care assist*
	Support	Care employee
	Attendant	Nurses aide
	RN	Nursing assistant
	EN	Personal care worker*
	Orderly	PCW
		AIN
		Assistant in nursing

Search terms were adapted for each database using appropriate controlled vocabulary and syntax.

### Study selection

Citations from each database were imported into Zotero (Zotero (Version 6.0.36), 2006) and exported to Covidence (2024). Title/abstract and full-text screening were conducted independently by two researchers using the predefined eligibility criteria. Discrepancies were resolved through discussion with a third researcher. Following completion of full-text screening, the reference lists of all studies meeting the inclusion criteria were screened for additional potentially eligible citations. Any citations identified during this process were manually added to Covidence and assessed using the same title/abstract and full-text screening process.

The searches identified 2379 records from electronic databases and 6 additional records through citation screening. Following removal of 835 duplicates, 1550 records underwent title and abstract screening, of which 1403 were excluded. Full texts were sought for 147 papers; 2 could not be retrieved, leaving 145 papers assessed for eligibility. Of these, 127 were excluded for the reasons shown in Figure 1, and 18 studies met the inclusion criteria. Of the six citation-screening records, three were removed as duplicates, two were excluded during title and abstract screening because they did not meet the eligibility criteria and one (MacDonald et al., 2006) was included in the final review.

**Figure 1.**
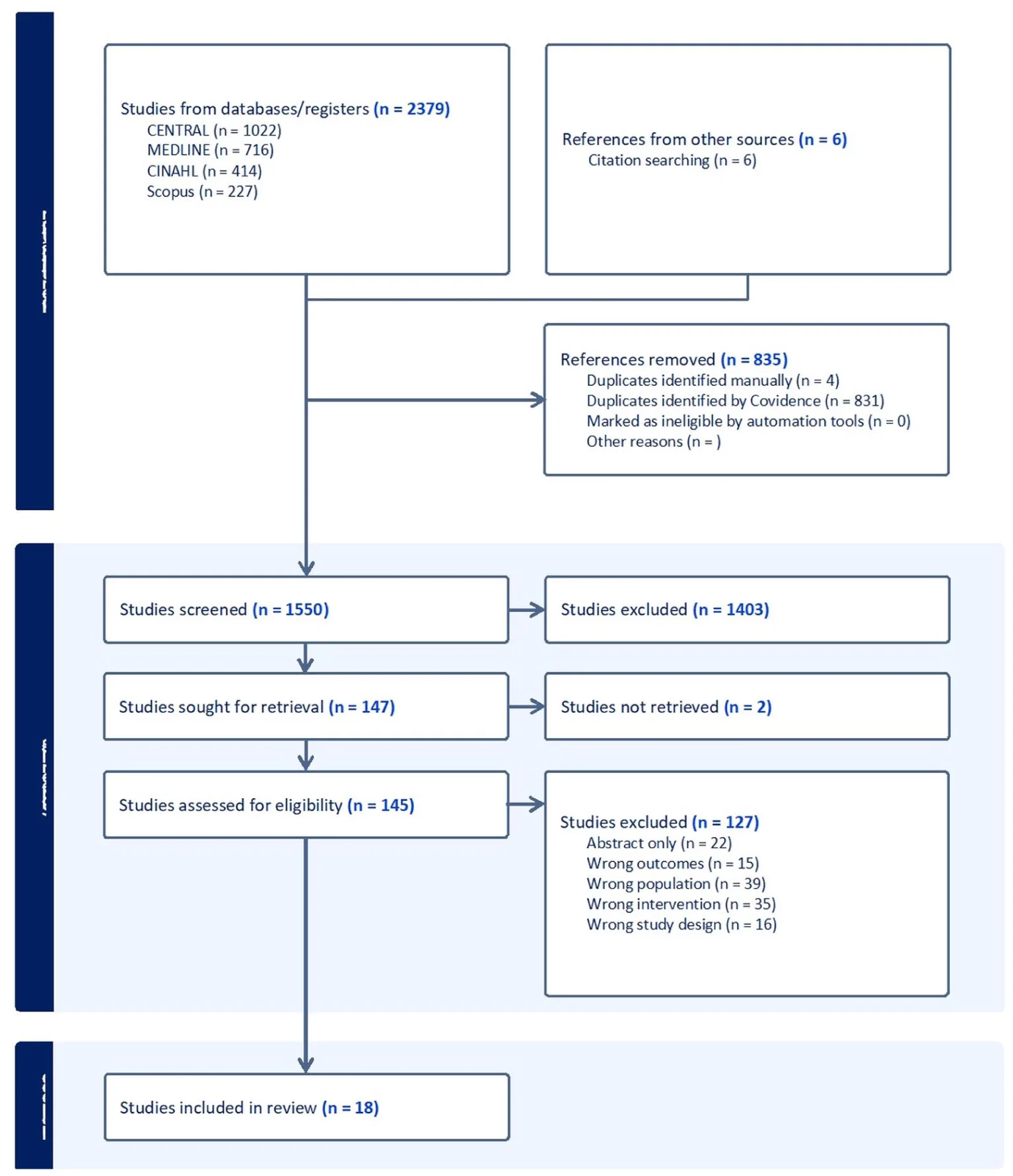
PRISMA diagram. PRISMA: Reporting followed the Preferred Reporting Items for Systematic Reviews and Meta-Analyses.

A PRISMA illustrative diagram of the search process is included below in Figure 1.

### Assessment of study quality

Methodological quality of the included studies was assessed using the Mixed-Methods Appraisal Tool (MMAT; Hong et al., 2018). Although critical appraisal is not mandatory for scoping reviews, it was undertaken to contextualise the strength and limitations of the heterogeneous evidence base and support cautious interpretation of the reported intervention outcomes. The MMAT enables the appraisal of qualitative, quantitative and mixed-methods studies within a single tool and was therefore appropriate for the diverse study designs included in this review. Two authors independently applied the relevant MMAT criteria, with discrepancies resolved through discussion (Hong et al., 2018). Consistent with MMAT guidance, individual item ratings were not aggregated into numerical scores; instead, studies were categorised as high, moderate, low or limited reporting quality based on response patterns. A narrative synthesis summarises MMAT findings, and MMAT qualitative judgements were reported separately within a result summary table (Table 3).

**Table 3.** Characteristics of included studies.

Authors (year); country	Population sample	Demographics	Programme name	Time to complete (approximate)	Quality assessment^a^
Online training modules					
Bentley et al. (2019)	*n* = 18	31–62 years	Recognising, diagnosing and managing dementia in general practice	Time: 3 h	MMAT: Limited reporting quality
Australia	Role/s: PNs in primary care	*M* = 45 years		Access: 4 weeks	GREET: Moderate
		Female: 17			
		Male: 1			
Bradford et al. (2023)	*n* = 43 (staff who completed post-training survey)	Not reported.	CARES^®^ Dementia Basics and CARES^®^ Dementia-Related Behavior^b^	Not reported	MMAT: Moderate
United States	Role/s: Nursing home staff (42% nurses, 35% CNAs, 23% not specified)				GREET: Moderate
Dobbs et al. (2018)	*n* = 48	*M* = 41.6 years	CARES^®^ ADL Dementia Care^b^	Time: 7–17 h	MMAT: Limited reporting quality
United States	Role/s: CNAs within the 10 recruited nursing homes	Female: 95%		Access: not reported	GREET: Moderate
Gallant et al. (2022)	*n* = 65 (post-training knowledge test sample)	Age not reported (overall)	No title – ‘online pain assessment training programme’^b^	Time: ~2 h total (modules 10–15 min each)	MMAT: Moderate
Canada	Role/s: Nurses and care aides working within the 7 LTC facilities recruited	Female: predominantly female		Access: 24 weeks	GREET: Moderate
Gaugler et al. (2016)	*n* = 40	*M* = 36.35 years	CARES^®^ Dementia-Related Behavior™ Online Training Programme^b^	Time: 4 h	MMAT: Moderate
United States	Role/s = DCWs working in nursing homes and assisted living facilities across 8 states	Female: 90%		Access: 4 weeks	GREET: Moderate
Goodenough et al. (2020)	*n* = 161 (KT follow-up survey respondents)	Not reported	Bedtime to Breakfast^b^	Time: 2 h	MMAT: Moderate
Australia	Role/s: Nurses, PCAs and other healthcare staff who completed the course Mar–Oct 2018			Access: 2 weeks	GREET: Moderate
Hobday et al. (2010b)	*n* = 40	21–63 years	CARES^®b^	Time: 3 h	MMAT: Moderate
United States	Role/s: CNAs working in one of the 5 care facilities recruited	Female: 35		Access: not reported	GREET: Limited
		Male: 5			
Hobday et al. (2010a)	*n* = 34	21–63 years	Internet-based multimedia education (IBME): CARES^®b^	Time: 2.2 h	MMAT: Moderate
United States	Role/s: DCWs working in a nursing home setting	Female: 85.3%		Access: not reported	GREET: Moderate
		Male/unknown: not reported			
Irvine et al. (2013)	*n* = 25	Age not reported	No title – ‘Internet dementia-training programme’^b^	Time: 2 h	MMAT: Moderate
United States	Roles: nurses/LPNs working within one LTC facility	Female: 85.9%		Access: 2 weeks	GREET: Moderate
		Male: 14.1%			
Levinson et al. (2025)	*n* = 50	18–64 years	Dementia Foundations Programme^b^	Time: 4 h	MMAT: Moderate
Canada	Role/s: Unregulated care providers, including PSWs and unregulated care companions) working in LTC or community care	Gender not reported		Access: 6 weeks	GREET: Moderate
MacDonald et al. (2006)	*n* = 95	22–64 years	Online Dementia Care Programme^c^	Time: 16 h	MMAT: Moderate
Canada	Role/s: Healthcare workers and professionals within the six recruited care facilities	Gender not reported		Access: 8 weeks	GREET: Moderate
McDermid et al. (2022)	*n* = 16	*M* = 51.2 years	Optimised WHELD Digital Learning Programme (eWHELD)	Time: 100–150 min	MMAT: Moderate
United Kingdom	Role/s: Staff within 16 nursing homes recruited: Care managers, nurses, senior care assistants, care assistants, care officers and other staff	Female: 14		Access: 8 weeks	GREET: Moderate
		Male: 2			
Pleasant et al. (2017)	*n* = 51	*M* = 51 years	CARES^®b^	Time: 4 h	MMAT: High
United States	Role/s: formal caregivers	Female: 45		Access: not reported	GREET: Moderate
		Male: 4			
Williams et al. (2021)	*n* = 219	*M* = 42.6 years	Changing Talk Online (CHATO)^d^	Time: 3–3.5 h	MMAT: Moderate
United States	Role/s: CNAs (*n* = 112), RNs (*n* = 55), LPNs (*n* = 17) working within 7 nursing homes recruited. Role description other/missing (*n* = 35) but are still formal caregivers	Female: 203		Access: not reported	GREET: Moderate
		Male: 15			
Yeh et al. (2023)	*n* = 92	*M* = 69 years	No title – ‘online training’^b,e^	Time: 35 h	MMAT: Moderate
United States	Role/s: IHSS caregivers	Female: 93%		Access: 10 weeks	GREET: Moderate
Microlearning training					
Aicken et al. (2021)	*n* = 25	Not reported	AWTD^f^	Time: 0.5 h	MMAT: High
United Kingdom	Role/s: HCAs working within the care homes recruited			Access: not specified, self-paced	GREET: Moderate
Jung et al. (2025)	*n* = 24	*M* = 62.17 years	No title – ‘YouTube-based mealtime assistance education programme for dementia care’^b^	Time: 49 min	MMAT: Moderate
South Korea	Role/s: DCWs working within the 2 LTC facilities recruited	Female: 24		Access: 7 days	GREET: High
		Male: 0			
Tompkins et al. (2020)	*n* = 25	20–69 years.	Mason music and memory initiative (M^3^I)^b^	Time: 1 h	MMAT: Moderate
United States	Role/s: DCWs working within the 2 recruited LTC communities	Female: 24		Access: 4 weeks	GREET: Moderate
		Male: 1			

When multiple sample sizes were reported, the population sample reflects the participants included in the outcome analysis.

a Full MMAT and GREET tables available in supplementary documents.

b Device required is any computer or device with internet access and browser.

c Computer with WebCT system loaded.

d Computer-only (explicitly not compatible with smartphone/tablet).

e The platform is not specified but is assumed to be an online training module.

f Smartphone and provided headset for the simulation experience.

PNs: Practice Nurses; MMAT: Mixed-Methods Appraisal Tool; GREET: Guideline for Reporting Evidence-Based Practice Educational Interventions and Teaching; CARES^®^: ‘Connect with the resident, Assess behavior, Respond appropriately, Evaluate what works, Share with the team’; CNAs: certified nursing assistants; ADL: activities of daily living; LTC: long term care; DCWs: direct care workers; IBME: Internet-based multimedia education; PCA: personal care assistant; PSW: personal support worker; eWHELD: Electronic Well-being and Health for people with Dementia; RN: registered nurse; LPN: licensed practical nurse; IHSS: in-home supportive services; HCAs: health care assistants.

The MMAT assessment identified variation in methodological quality across the included studies. Fourteen studies were assessed as having moderate methodological quality, two as having high methodological quality (Aicken et al., 2021; Pleasant et al., 2017) and two as having limited reporting quality (Bentley et al., 2019; Dobbs et al., 2018). Common strengths included clear research questions and alignment between the research aims and study methods. However, some quantitative studies were limited by small sample sizes, which reduced statistical power. Five studies incorporated both qualitative and quantitative components (Goodenough et al., 2020; Hobday et al., 2010a, 2010b; MacDonald et al., 2006; Tompkins et al., 2020). None explicitly described a mixed-methods framework or clearly articulated how the qualitative and quantitative findings were integrated. Hobday et al. (2010b) also provided limited methodological detail regarding study design and statistical reporting. Critical appraisal informed interpretation of the evidence rather than eligibility; therefore, despite variation in methodological rigour, all 18 studies were retained.

The Guideline for Reporting Evidence-based Practice Educational Interventions and Teaching (GREET) checklist (Phillips et al., 2016) was used to assess the completeness of reporting of educational interventions. Although GREET does not prescribe a total score or grading system, deriving qualitative summary categories can facilitate transparent comparison of reporting completeness across studies in a scoping review. Accordingly, overall reporting completeness for each intervention was categorised as high, moderate or limited based on the proportion and adequacy of GREET items reported. To summarise GREET findings, a narrative approach was adopted, whereas qualitative judgements were presented separately in the results summary table (Table 3). The GREET appraisal identified moderate reporting completeness in 16 studies, high completeness in one and limited completeness in one (Table 3). Intervention content, educational materials and delivery mode were consistently reported, whereas information concerning instructors, content-developer expertise, engagement monitoring, intervention adaptation and fidelity was reported less consistently. No studies were excluded based on the GREET appraisal.

### Data charting and synthesis

One researcher created a data extraction table to collect study characteristics and outcomes related to the research questions. This researcher completed the initial data extraction, and a second researcher reviewed a 40% sample to verify accuracy. Extracted data were tabulated and included study details: author(s), title, year of publication, country of publication, study methodology, population type, sample size, intervention type and characteristics, quality assessment overview and key findings. The extracted data are presented in Table 3 and Table 4. When multiple sample sizes were reported (e.g. training completion, certification completion or survey response), the sample size reported in this review reflects the number of participants contributing outcome data.

**Table 4.** Outcome measures and key findings.

Study	Aim	Method; outcome measures	Key findings as identified by authors				
			Knowledge	Skills	Self-efficacy/confidence	Programme comments/suggestions	Training modality comments/recommendations
Aicken et al. (2021)	To examine the effectiveness of simulation-based training in enhancing dementia care skills to improve quality of life.	In-depth interviews (pre/post). 7-item post-training survey: 5 scaled items, 2 open-ended.	No reported change.	No reported change.	Participants reported no substantial improvement in confidence. Increased empathy and understanding of dementia experience (e.g. greater patience)	Nil	VR headsets caused disorientation and discomfort related to being observed. AWTD was described as quick and easy to deploy, with minimal setup, and was recommended for dementia staff orientation.
Bentley et al. (2019)	To evaluate the impact of an online educational resource on the knowledge, attitudes, confidence and behavioural intentions of primary care professionals.	27 item DKAS pre and post-training. 15 item GPACS-D using ranking scale questions 12 item CPD reaction questionnaire Semi-structured interviews.	28% increase in knowledge post-training.	PNs reported improved skills in using a systematic approach to working with people with dementia.	PNs showed a 16% increase in confidence/attitudes (GPACS-D). Interviews indicated greater confidence in identifying and managing dementia.	78% completion among PN participants. Time required to complete modules limited completion within the evaluation period.	Authors highlighted the online format as a strength for rural and remote primary care professionals with limited dementia care resources.
Bradford et al. (2023)	To evaluate the effect of CARES^®^ dementia training programme on reducing use of antipsychotic medication, as well as to assess staff satisfaction, learning outcomes and behavioural changes post-training.	Run chart analysis of antipsychotic use (6 months pre/post-training). Likert-scale satisfaction survey. Post-training CARES^®^ module exams. Free-text surveys assessing care behaviour change.	Mean post-training CARES^®^ Dementia Basics exam score: 89% (no pre-training comparison).	Staff reported applying CARES^®^ approach behaviours in practice (e.g. communication, behaviour assessment, care adaptation).	97% agreed/strongly agreed they were more confident in dementia care skills post-programme. 91% agreed/strongly agreed the videos gave them new ideas on how to interact with people with dementia.	97% agreed/strongly agreed they would recommend the programme to other nursing home workers.	Internet-based training was viewed as more engaging than traditional classroom or reading-based methods. Most staff found the training content easy to understand and follow. Graphics, sound and video enhanced engagement. Authors noted the CARES^®^ programme may be cost-prohibitive without grant funding.
Dobbs et al. (2018)	To evaluate the implementation of a CARES^®^-based online training.	Nine Likert-scale and 5 open-ended items.	90% agreed/strongly agreed the programme provided new dementia care ideas. CNAs reported gaining new dementia care knowledge not previously known.	CNAs reported learning practical daily care techniques (e.g. bathing, feeding, maintaining dignity). Training supported more reflective care approaches and use of the CARES^®^ framework for managing challenging situations.	90% agreed/strongly agreed they were more confident in dementia care skills post-training. 85% agreed/strongly agreed they felt more confident and comfortable communicating with and caring for people with dementia. Thematic analysis indicated increased confidence.	Video clips showing real-life care supported links between theory and practice. Module length viewed as excessive. Participants reported that video content was perceived as monotonous, repetitive and overly lengthy. Suggestions from participants included fewer, shorter videos, more interactivity, quizzes, humour and family perspectives. Greater use of interactive knowledge checks and quizzes was recommended to enhance learning.	Online, interactive format valued as convenient, easy to use and self-paced. Technical issues (e.g. buffering, navigation, auto-timeout) were reported and subsequently resolved. Authors recommend time-saving strategies to support person-centred care in high-workload settings.
Gallant et al. (2022)	To develop and evaluate an interactive online training programme to improve pain assessment for residents with dementia and support remote implementation of a standardised protocol in rural long-term care facilities.	14-item pain (KT) scale (pre/post). 10-item RFOC scale. 31-item OTEQ. Facility-level pain quality indicators. Semi-structured staff interviews.	Significant increase in pain assessment knowledge (median score increased from 71 to 93, *p* < 0.001)	Improved pain assessment practices (e.g. increased frequency of admission and weekly assessments), though follow-up actions were less consistently improved.	Confidence not measured using a validated scale; Qualitative findings indicated improved confidence and perceived competence in pain assessment practices post-training and protocol implementation.	Online training and standardised protocol well received by staff. Implementation barriers: time constraints, competing demands, organisational variability. Recommend: use of implementation science frameworks (e.g. CFIR) to support sustained practice change.	Remote online training and protocol implementation were feasible in rural LTC facilities. Online delivery addressed geographic barriers to continuing education in rural settings. Need for ongoing support and site-specific adaptation to maximise digital training impact.
Gaugler et al. (2016)	To evaluate if CARES^®^ Dementia-Related Behavior™ online programme improves direct care workers’ knowledge for responding to dementia-related behaviours and assess programme acceptability and perceived usefulness.	Dementia care knowledge: CARES^®^ knowledge test (25 items; MCQ/true–false; pre/post). Programme acceptability/utility: Post-training questionnaire (satisfaction, usability, perceived practice impact; open-ended).	Dementia-related behaviour knowledge significantly increased post-training compared with pre-training (*p* < 0.01).	Skills not objectively assessed; Participants reported applying learned strategies in practice post-training.	Confidence not measured using a validated scale; Descriptive responses indicated increased perceived ability to respond to dementia-related behaviours.	High satisfaction with programme content and real-world relevance. Authors recommended CARES^®^ Dementia-Related Behavior™ as a feasible and acceptable dementia behaviour education approach for direct care workers. Real-person video scenarios enhanced realism and engagement.	Online, self-paced delivery was viewed as practical for staff with limited time and variable schedules. Online delivery reduced barriers associated with in-person training, particularly staffing and time constraints.
Goodenough et al. (2020)	To evaluate the impact of a short online course on dementia care knowledge.	PIQOC (single-item, positively packed Likert scale) 10-item yes/no survey generating KT score.	Total sample KT score was significant (*p* < 0.001); Formal caregiver-specific analysis not reported.	Not measured.	Formal caregiver-specific PIQOC outcomes were not analysed separately.	Over 90% course completion rate.	Nil
Hobday et al. (2010a)	To develop a mobile-friendly online dementia training programme for direct care workers in nursing homes, improving accessibility.	Post-programme feasibility measure (20 items; 5-point Likert). 5 additional open-ended items. Knowledge MCQ (15 items; pre/post).	Over 80% showed significant post-test knowledge gains (*p* < 0.0001). DCWs reported improved understanding of dementia care.	Not measured.	All participants reported increased confidence in dementia care skills post-training.	Nil	Online training was accessible, but participants requested hardcopy resources. Authors noted greater flexibility than face-to-face training and recommended larger controlled studies.
Hobday et al. (2010b)	To evaluate the CARES^®^ training programme’s effectiveness in enhancing dementia care knowledge among CNAs.	Dementia care knowledge MCQ (15 items; pre/post; reordered). CARES post-test evaluation survey (19 items; 5-point Likert). 4 open-ended post-test items.	78% CNAs showed knowledge gains pre/post (*p* = 0.013) Participants reported improved dementia care understanding and confidence in peer communication.	Over 80% self-reported skill improvement.	Over 80% self-reported increased confidence.	Nil	The online format enabled staff to train at their convenience.
Irvine et al. (2013)	Evaluate whether an internet-based dementia training programme, originally developed for nurse aides, improves dementia-related knowledge, self-efficacy, attitudes and behavioural intentions among nursing staff and non-direct care workers in long-term care.	Dementia knowledge: VSTs assessing appropriate care responses. Self-efficacy: VST-based ratings for managing dementia-related situations. Psychosocial outcomes: Attitudes towards dementia care and behavioural intentions. Programme acceptability: Post-training acceptability and perceived usefulness.	Dementia care knowledge significantly improved post-training, with small-to-moderate effect sizes.	Skills not directly observed; Improvements inferred from increased behavioural intentions post-training.	Self-efficacy for managing dementia-related situations significantly improved post-training, with small-to-moderate effect sizes.	Training was acceptable across a broad range of staff roles. Authors recommended cross-training multiple staff groups using a single dementia-focused online programme.	Internet-based delivery enabled flexible access across shifts and staff roles. Low reading level, no keyboarding and self-paced navigation supported usability across varying computer literacy. Internet training conserved organisational resources while expanding access to dementia education.
Jung et al. (2025)	Evaluate the feasibility and effectiveness of a nurse-led, YouTube-based online video education programme to improve dementia knowledge, attitudes and care self-efficacy related to mealtime assistance among long-term care workers.	DKAT2 (21 items). ADQ (19 items). Care Self-Efficacy Scale (30 items; mealtime care, eating behaviour problems, communication). 7-item post-training satisfaction questionnaire.	Dementia knowledge significantly increased post-training (*p* < 0.001).	Skills not directly assessed using observational or performance-based measures.	Care self-efficacy significantly improved post-training (*p* < 0.001), across all subdomains.	Participants reported high satisfaction with the education programme. Programme was acceptable and engaging for long-term care workers and suitable for dementia-specific mealtime education. Authors cautioned that improvements in dementia attitudes should not be conflated with gains in knowledge or care skills.	Short YouTube-based videos supported flexible, self-paced learning. Microlearning video format was feasible for shift-based care staff. Video-based platforms were recommended as a scalable approach for dementia education in long-term care.
Levinson et al. (2025)	Evaluate the usability, acceptability and impact of a web-based dementia education programme (Dementia Foundations Programme) on dementia knowledge, attitudes and confidence among unregulated care providers.	DKAS (pre/post). DAS (pre/post). DCCS (pre/post). SUS. Post-programme evaluation questionnaire.	DKAS scores significantly improved post-training.	Skills not directly assessed using observational or performance-based measures.	DCCS significantly improved post-programme.	Dementia attitudes (DAS) significantly improved post-training. Participants reported high satisfaction with programme content and delivery. Authors recommended the Dementia Foundations Programme acceptable and scalable option for unregulated care providers.	Web-based, self-paced delivery supported flexible access and completion. Online delivery was suitable for broad workforce dissemination across varied schedules and training backgrounds. Programme usability rated good to excellent (SUS).
MacDonald et al. (2006)	To develop an online dementia training programme for healthcare workers and assess its impact on care quality.	21-item pre-programme survey. 22-item ‘temperature check’ (20 × 4-point Likert; 2 open-ended). 45-item post-programme survey (41 × 4-point Likert; 4 open-ended). In-depth interviews Programme records reviewed for learner experience and utilisation data. Email interviews with facilitator and instructional designer.	Interviews with nurse aides/HCAs suggested improved knowledge, understanding of dementia-related behaviours (incl. aggression) and shifts in resident perceptions.	Interviews suggested nurse aides/HCAs adopted different interaction approaches post-training.	Not measured.	Some learners spent 5–6 h online weekly, exceeding estimates. Programme language level was perceived as too advanced.	Some participants experienced computer literacy challenges. Flexible access was valued; authors recommended more user-friendly design with adequate computer support.
McDermid et al. (2022)	Compare the effectiveness of a digital adaptation of the WHELD programme with virtual coaching support versus digital eWHELD alone in improving staff outcomes and resident well-being in care homes.	ADQ (19 items). KIDE (16 items). DCM (resident well-being and engagement). Engagement and delivery fidelity (descriptive).	No significant difference in dementia knowledge between supported and unsupported eWHELD groups at follow-up.	Staff skills not directly assessed; practice change inferred from resident-level outcomes (DCM).	Not measured using a self-efficacy or confidence scale.	Engagement with intervention varied across sites.	Digital delivery was feasible, but effectiveness improved with virtual coaching and support. Dementia care attitudes (ADQ) significantly more positive in supported eWHELD group than unsupported digital-only group, and residents in supported eWHELD group showed higher well-being and greater engagement in positive activities than unsupported group. Authors emphasised importance of support mechanisms (e.g. coaching) to maximise digital training effectiveness.
Pleasant et al. (2017)	To evaluate the effectiveness of the CARES^®^ Dementia Basics programme in enhancing dementia care knowledge and understanding.	16-item dementia knowledge MCQ (pre/post/follow-up). SCIDS Professionalism subscale (5 items). Video-scenario MCQs identifying person-centred care.	Dementia knowledge significantly improved post-test and at follow-up (*p* < 0.001); Formal caregivers showed higher knowledge, but analyses underpowered.	Not measured.	SCIDS subscale results for formal caregivers were not analysed separately.	One-time 4-h training may not sustain long-term improvements in dementia attitudes.	Nil
Tompkins et al. (2020)	To assess efficacy of the M3I microlearning programme in enhancing dementia care knowledge and understanding in direct care workers.	9-item 4-point Likert survey (pre/post); 2 open-ended post.	Statistically significant improvement.	Staff reported 30 cases of reduced unwanted resident behaviours post-programme.	Not measured for self-efficacy/confidence in dementia care.	M3I modules addressed dementia behaviour and person-centred care, but related outcomes were not assessed.	The programme was flexible for shift work.
Yeh et al. (2023)	To assess effectiveness of online dementia care training programme in enhancing IHSS caregivers’ knowledge and self-efficacy.	DKAT2 (21 items). Caregiver Self-Efficacy Scale (9 items; 2 domains; 1–10 scale). CSAQ (18 items; yes/no + 1–10 rating). PHQ-2 (2 items; 0–3 scale).	DKAT2 scores significantly improved post-training and at follow-up.	Over 95% agreed/strongly agreed the training improved caregiving skills.	Self-efficacy in dementia management significantly increased post-training and at follow-up.	Over 90% of participants were very satisfied with the training.	Nil

VR: Virtual Reality; AWTD: A Walk Through Dementia™; DKAS: Dementia Knowledge Assessment Scale; GPACS-D: General Practitioner Attitudes and Confidence Scale for Dementia; CPD: Continuing Professional Development; PNs: Practice Nurses; CARES^®^: ‘Connect with the resident; Assess behavior; Respond appropriately; Evaluate what works; Share with the team’; CNAs: Certified Nursing Assistants; KT: Knowledge Test; RFOC: Readiness for Organisational Change; OTEQ: Online Training Evaluation Questionnaire; CFIR: Consolidated Framework for Implementation Research; LTC: Long-Term Care; MCQ: Multiple-choice question; DCWs: Direct Care Workers; PIQOC: Perceived Improvement in Quality of Care; PCA: Personal Care Assistant; VST: Video Situation Test; DKAT2: Dementia Knowledge Assessment Tool, Version 2; ADQ: Approaches to Dementia Questionnaire; DAS: Dementia Attitudes Scale; DCCS: Dementia Care Confidence Scale; SUS: System Usability Scale; HCAs: Health Care Assistants; KIDE: Knowledge in Dementia Scale; DCM: Dementia Care Mapping; eWHELD: Optimised Well-being and Health for People with Dementia Digital Learning Programme; SCIDS: Sense of Competence in Dementia Care Staff Scale; M3I: Mason Music & Memory Initiative; CSAQ: Care Staff Attitudes Questionnaire; PHQ-2: Patient Health Questionnaire–2; IHSS: In-Home Supportive Services.

For studies including multiple professional populations, only the isolated data for formal caregivers were extracted. However, programme-level findings and training modality descriptions were recorded as reported, even when not specific to formal caregivers. A narrative synthesis was conducted following Popay et al.’s (2006) approach to integrate findings across studies.

## Results

### Study characteristics

Studies were published between 2006 and 2025 and were conducted across 5 countries: the USA (*n* = 10), Canada (*n* = 3), Australia (*n* = 2), the United Kingdom (*n* = 2) and South Korea (*n* = 1). Most studies focused on staff in residential long-term care, although several included participants from community or mixed care settings.

Studies included between 16 and 219 participants, reflecting both small-scale evaluations and larger multi-site designs. Two pilot studies (Hobday et al., 2010b; Irvine et al., 2013) and one feasibility study (Hobday et al., 2010a) were included. Across studies, participants were predominantly female.

Study designs were heterogeneous. Ten employed primarily quantitative designs. One used a predominantly qualitative design, whereas the remaining seven studies adopted mixed-methods approaches that combined quantitative measures with qualitative interviews or implementation data. Terminology describing the formal caregiving workforce differed widely, including registered nurses, enrolled nurses, direct care workers, nurses’ aides, personal support workers, care aides, healthcare assistants and unregulated care providers.

### Types of digital training available

Structured online training modules represented the most common delivery format. Microlearning approaches, including short video-based programmes and brief modular content, appeared in only three. Training duration ranged from 1 h or less, 2–5 h to 7 h or more. One study did not report the training duration. All digital training interventions were delivered asynchronously.

### Outcomes measures

Outcomes were inconsistently defined, measured and reported across studies, with limited objective assessment of practice change.

Three studies assessed attitudes towards dementia using validated instruments. McDermid et al. (2022) and Jung et al. (2025) used the Approaches to Dementia Questionnaire, whereas Levinson et al. (2025) used the Dementia Attitudes Scale. Although informative, these measures reflected beliefs rather than skill or competence and were secondary to the review’s aims.

### Impact on knowledge

Knowledge was assessed in all 18 studies using various approaches, including internally developed quizzes, validated dementia knowledge instruments and qualitative interviews exploring perceived learning. Knowledge domains commonly focused on dementia symptoms, behavioural management, communication approaches and task-specific care (e.g. pain or mealtime care).

Improvements in dementia-related knowledge following digital training were reported across studies using a range of assessment approaches. Three studies used internally developed pre–post knowledge measures and reported statistically significant improvements. Gaugler et al. (2016) reported higher post-training knowledge scores (*p* < 0.01). Hobday et al. (2010b) similarly reported a significant increase (*p* = 0.013), as did Hobday et al. (2010a; *p* < 0.001). MacDonald et al. (2006) used descriptive survey data and qualitative interviews, which indicated improved understanding of dementia and dementia-related behaviours but did not provide a statistical test of knowledge change. Improvements measured using validated instruments were reported by Levinson et al. (2025), using the Dementia Knowledge Assessment Scale, and by Yeh et al. (2023) and Jung et al. (2025), using the Dementia Knowledge Assessment Tool Version 2, whereas three others assessed knowledge qualitatively or through descriptive survey items, reporting perceived increases in understanding following training (Aicken et al., 2021; Gallant et al., 2022; Tompkins et al., 2020). An additional three studies reported improvements using structured pre–post knowledge assessments; however, results for formal caregivers could not be isolated from the larger sample of health professionals (Bradford et al., 2023; Dobbs et al., 2018; Pleasant et al., 2017).

### Impact on skills

Skills were inconsistently measured and rarely assessed with objective or observational methods. In the 10 studies where skill outcomes were examined, they were most often evaluated through self-reported perceptions of skill improvement, descriptive survey items or qualitative accounts of changes in care approaches. One study documented practice-level changes, reporting improvements in the frequency and timeliness of pain assessment following protocol implementation (Gallant et al., 2022). No study employed validated observational tools to directly assess dementia care skills.

Skills-related outcomes were assessed in 10 of the 18 studies. Skills were assessed either through self-reported perceptions of caregiving ability or through qualitative descriptions of perceived changes in care practice. Reported improvements included communication, care approaches and recognition of resident needs. No included study used objective or observational measures to directly assess skill acquisition or behavioural change at the individual caregiver level. One study reported facility-level reductions in dementia-related behaviours, although these outcomes could not be attributed to individual caregiver skill development (Tompkins et al., 2020).

### Impact on self-efficacy

Confidence or self-efficacy outcomes were reported in 11 of the 18 included studies. Validated measures included the General Practitioner Attitudes and Confidence Scale for Dementia (Bentley et al., 2019); the Dementia Care Confidence Scale (Levinson et al., 2025); the Caregiver Self-Efficacy Scale (Yeh et al., 2023) and the Care Self-Efficacy Scale (Jung et al., 2025). Irvine et al. (2013) assessed self-efficacy using scenario-based Video Situation Tests. The remaining studies used internally developed survey items or qualitative interview approaches to assess perceived competence or preparedness for care delivery. Although Goodenough et al. (2020) reported confidence outcomes using the Perceived Improvement in Quality of Care survey, data specific to formal caregivers were not reported separately.

Improvements in confidence or self-efficacy were observed, with outcomes typically assessed using pre–post designs. These improvements were reported in relation to both general dementia care and specific care activities, such as mealtime assistance.

Digital training was generally associated with reported improvements in confidence or perceived competence in providing dementia care. However, outcome measurement approaches varied substantially, and most studies relied on self-reported perceptions of confidence or competence rather than objective or observational measures of behavioural change.

### Identified advantages of digital training

Flexibility and accessibility were the most frequently cited advantages of digital dementia training across the included studies. Formal caregivers valued the ability to complete training at convenient times and places, which suited the realities of shift work, irregular schedules and staffing pressures. One study noted that digital delivery was a practical solution to barriers associated with accessing face-to-face education, enabling participation outside scheduled shifts and reducing the perceived impact on staff time (Tompkins et al., 2020).

Ease of implementation and delivery was noted in two studies (Aicken et al., 2021; Dobbs et al., 2018). Gallant et al. (2022) highlighted that digital training could address geographic barriers to training in remote areas. The microlearning approach was described as ‘quick and easy to deploy’, supporting scalability across organisations (Aicken et al., 2021: 16). Additional benefits of the digital format were noted across five studies: consistent training content (Dobbs et al., 2018), the ability to revisit training materials and positive staff acceptability (Bentley et al., 2019; McDermid et al., 2022). Interactive or brief modular content was especially well received, with learners reporting enhanced usability and engagement (Jung et al., 2025; Levinson et al., 2025).

In addition, several studies highlighted the feasibility and acceptability of digital training, suggesting it can be readily implemented within real-world care settings.

### Challenges of digital training

Despite the advantages, several challenges associated with digital dementia training were identified (as detailed in Table 4). These challenges were primarily related to training design, technological requirements, time constraints and workforce context, rather than to the digital format itself.

Workforce and organisational constraints were the most reported barriers. Three studies noted that staff lacked protected time during work shifts to complete training or faced competing workplace demands that limited engagement (Aicken et al., 2021; Gallant et al., 2022; MacDonald et al., 2006). Staff reported that training needed to be completed outside paid work hours or alongside existing workloads, which reduced participation or completion rates (Aicken et al., 2021). Organisational variability and competing priorities were also identified as barriers to implementing digital dementia training in routine practice (Gallant et al., 2022).

Challenges related to training design were also identified. In two studies, the length or structure of digital modules affected engagement. Dobbs et al. (2018) found that longer modules or repetitive video content were viewed as burdensome, with participants recommending shorter segments, greater interactivity and varied learning materials. Similarly, Bentley et al. (2019) reported that the time required to complete modules limited the number of completions during the evaluation period. Language complexity was reported as a barrier by MacDonald et al. (2006) who found that participants perceived the programme content as written at a level too advanced for some learners. In addition, Pleasant et al. (2017) reported that a single-session digital intervention may be insufficient to sustain longer-term changes in dementia-related attitudes.

Technological usability barriers were reported less frequently but remained relevant in some contexts. For example, participants in immersive virtual reality training reported discomfort or disorientation while wearing headsets (Aicken et al., 2021). Furthermore, technical issues such as video buffering, navigation challenges or automatic session timeouts were reported during early implementation of online modules (Dobbs et al., 2018). Limited computer literacy among some staff was also identified as a barrier to engagement with digital training platforms (MacDonald et al., 2006).

Implementation considerations were also highlighted in two studies. McDermid et al. (2022) noted that digital-only training programmes may require additional support mechanisms, such as coaching or organisational facilitation, to achieve meaningful practice change. Cost considerations were raised by Bradford et al. (2023), who noted that access to certain digital training programmes may be limited without external funding support.

## Discussion

This review examined the digital dementia training platforms available to formal caregivers, their effects on knowledge, skills and self-efficacy and whether learning outcomes translated into changes in caregiving behaviour. The findings are discussed below in relation to each research question and the wider literature, followed by implications for the design and implementation of future dementia training.

### Digital training platforms available to formal caregivers

The digital dementia training platforms identified in this review predominantly comprised structured asynchronous online modules, whereas microlearning approaches were examined in only three studies. This finding is consistent with the predominance of asynchronous online programmes identified in an earlier review of technology-delivered dementia education (Scerbe et al., 2019). Although Scerbe et al. (2019) systematically reviewed pre–post evaluations among healthcare providers, the present scoping review mapped evidence from diverse study designs across the broader formal-caregiver workforce and specifically examined whether learning outcomes translated into caregiving behaviour. Although microlearning remains comparatively underexplored in dementia care, its brief and focused format may be particularly suited to formal caregivers working in time-constrained environments, consistent with findings from the broader health-professions education literature (De Gagne et al., 2019).

Digital dementia training offers practical advantages that support its relevance for the formal caregiving workforce. Flexibility and accessibility were reported as benefits in several studies, enabling caregivers to engage with training outside of traditional time and location constraints, which is an important consideration in shift-based and resource-constrained care environments (Hobday et al., 2010a, 2010b; MacDonald et al., 2006; Tompkins et al., 2020). Digital platforms also facilitate consistent delivery of content and repeated access to learning materials, which may reinforce knowledge over time (Bentley et al., 2019; Dobbs et al., 2018; McDermid et al., 2022). The three microlearning studies reported promising findings for caregiver knowledge and confidence, although the small evidence base limits conclusions about its comparative effectiveness.

### Influence on knowledge, skills and self-efficacy

Digital dementia training was generally associated with improvements in dementia-related knowledge and perceived self-efficacy. However, caregiving skills were assessed less frequently and were predominantly evaluated through self-reported or qualitative outcomes. This pattern is consistent with broader digital health education research, in which online training commonly improves knowledge and self-efficacy, whereas evidence concerning skill development remains less consistent (Roberts et al., 2024; Scerbe et al., 2019; Wu et al., 2024).

Interpretation of these findings must also consider limitations in outcome measurement and study quality. Although improvements in knowledge, skills and self-efficacy were frequently described, these outcomes were not always clearly differentiated. In several studies, participants reported increased ‘understanding’ of dementia or improved approaches to care, which may reflect a combination of cognitive, affective and behavioural changes rather than discrete constructs (Aicken et al., 2021; Gallant et al., 2022; Tompkins et al., 2020). Reliance on self-reported outcomes, variability in methodological quality and incomplete reporting of educational interventions, further limit comparisons across studies. Inconsistent reporting of theoretical underpinning, intervention development and implementation processes restricts the assessment of content validity and fidelity, reducing confidence in conclusions regarding the effectiveness of digital dementia training.

### Translation of learning into caregiving behaviour

Evidence that learning translated into changes in caregiving behaviour was limited. No studies used validated direct observational measures to demonstrate that improvements in knowledge, skills or self-efficacy translated into changes in individual caregiving practice. Where behavioural change was reported, it was generally inferred from participants’ perceptions, qualitative accounts or indirect practice-level outcomes rather than direct observation. This finding is consistent with broader digital health-professions education research, which has similarly identified limited evaluation of behavioural and practice-level outcomes following digital training (De Gagne et al., 2019; Scerbe et al., 2019).

Although some studies described perceived or practice-level changes following training (Aicken et al., 2021; Gallant et al., 2022; Tompkins et al., 2020), the absence of validated direct observational assessment limits confidence that learning translated into individual caregiving behaviour. In addition, only one study examined longer-term outcomes, such as knowledge retention or sustained changes in practice over time (Pleasant et al., 2017). Together, these gaps restrict conclusions regarding the durability and real-world impact of digital dementia training interventions.

### Implications for training design and implementation

These findings have important implications for dementia-care workforce education, implementation, policy and future research. Globally, an estimated 142 million older adults are unable to meet basic needs independently, and approximately two-thirds will require formal care (World Health Organization, 2022). This demand places increasing pressure on long-term care and healthcare systems and highlights the critical role of formal caregivers, including nurses, personal care workers and nursing assistants, in delivering effective dementia care. Ensuring this workforce has access to scalable, effective and accessible training is essential for supporting care quality and workforce preparedness.

A critical gap identified in this review relates to the underrepresentation of direct care workers, who provide the majority of hands-on support for people living with dementia. These roles, including personal care workers, nursing assistants and other unregulated care providers, are primarily responsible for activities of daily living, ongoing monitoring and day-to-day care management (Australian Institute of Health and Welfare, 2024). Despite this central role, few studies examined outcomes for direct care workers as a distinct group, with results frequently aggregated across heterogeneous formal caregiver samples that included regulated nursing staff. This limits understanding of how digital dementia training addresses the specific training needs and workplace contexts of frontline caregivers. Future digital training should therefore be designed and evaluated specifically with direct care workers to ensure its content, delivery and outcomes reflect their needs.

Digital dementia training platforms, including microlearning approaches, offer practical advantages due to their flexible formats, which can be delivered across diverse care settings and workforce groups. These platforms can reach geographically dispersed workforces and reduce barriers associated with traditional face-to-face training. The scalability of digital training enables expanded access to dementia education; however, successful implementation will depend on organisational support, workforce capacity and the ability to integrate training within existing care environments. Training design should therefore prioritise brief or modular content, accessible language, interactive learning activities and opportunities to revisit educational materials.

Despite these advantages, implementation challenges remain. Limited computer literacy, discomfort with unfamiliar technologies and a lack of protected time during work hours were reported as barriers (Aicken et al., 2021; Gallant et al., 2022; MacDonald et al., 2006). In one study, caregivers reported completing training outside paid work time, potentially reducing engagement and uptake (Aicken et al., 2021). These findings align with broader digital health education research, highlighting organisational support and workload pressures as key determinants of successful adoption of digital training in healthcare settings (De Gagne et al., 2019; Haghighat et al., 2023). Caregivers’ experiences with digital training modalities, including acceptability, usability and engagement, were also underexplored (Gallant et al., 2022; McDermid et al., 2022; Tompkins et al., 2020). Future implementation studies should assess these factors and consider the provision of protected work time, technical assistance and other organisational supports needed to facilitate participation.

Despite promising outcomes, evidence remains limited regarding the extent to which digital training translates into sustained changes in practice or measurable improvements in care quality. Future research should incorporate objective measures of skill acquisition, behavioural change and care-related outcomes, alongside longer-term follow-up, to better understand real-world impact of digital dementia training. Evaluations should also clearly report the theoretical basis, development, implementation and fidelity of training interventions to support interpretation and replication.

### Strengths and limitations

The findings should be interpreted in light of the methodological strengths and limitations of this review. This review employed a rigorous and comprehensive search strategy across multiple databases, using broad search terms to capture the range of digital dementia training interventions available to formal caregivers. The inclusion of diverse methodologies allowed for a comprehensive mapping of the current evidence base, consistent with the objectives of a scoping review. Application of the MMAT and GREET tools provided structured assessment of methodological and reporting quality.

However, several limitations should be noted. Substantial variability across studies in intervention design, outcome measures and reporting limited direct comparison between studies. Inconsistent use of knowledge, skills and self-efficacy assessment tools further complicated the interpretation of results. Many studies also relied on self-reported outcomes, with limited use of objective behavioural or care-related measures. Studies that incorporated blended or face-to-face training components were excluded where digital-specific outcomes could not be isolated, which may have reduced the number of eligible studies and limited the generalisability of the findings. Finally, relatively few studies focused specifically on direct care workers, limiting the generalisability of findings to this high-priority group.

## Conclusions

Digital dementia training for formal caregivers shows promise for improving knowledge and self-efficacy and offers a flexible, scalable approach to workforce education. This review demonstrates that structured asynchronous modules currently predominate, whereas microlearning represents an emerging but underexplored approach. It also identifies a consistent gap between improvements in learning outcomes and demonstrated practice change, establishing objective assessment of skills and caregiving behaviour as a priority for future evaluation.

Future research should prioritise rigorous evaluation of behavioural and care-related outcomes, alongside longer-term follow-up and a greater focus on frontline caregiving populations. Effective digital dementia training should combine brief, accessible and interactive content with organisational support, protected training time and technical assistance. Training should also be designed and evaluated specifically with direct care workers to ensure relevance to those providing most hands-on dementia care. Strengthening the evidence base in these areas will support the development of effective and accessible training approaches and contribute to improved dementia care.

**Key points for policy, practice and research**

- Digital dementia training was associated with improvements in formal caregivers’ dementia-related knowledge and self-efficacy across residential, community and healthcare settings.
- Flexible online and microlearning approaches may support nursing practice by improving access to dementia education for shift-based and geographically dispersed workforces.
- Evidence for changes in caregiving skills and behaviours remains limited, with few studies using objective measures of practice change or care quality outcomes.
- Health and social care policy should prioritise accessible, scalable dementia education and support further research into long-term workforce and care-related outcomes.
- Further research should prioritise objective measures of behavioural change and care-related outcomes, longer-term follow-up and greater representation of direct care workers.

## Supplemental Material

sj-docx-1-jrn-10.1177_17449871261495844 – Supplemental material for Digital dementia training for formal caregivers: a scoping review of platforms, outcomes and evidence gaps Supplemental material, sj-docx-1-jrn-10.1177_17449871261495844 for Digital dementia training for formal caregivers: a scoping review of platforms, outcomes and evidence gaps by Clare Barrett, Wendy Moyle, Jonathan Foo, Marie-Claire O’Shea and Lana Mitchell in Journal of Research in Nursing

sj-xlsx-1-jrn-10.1177_17449871261495844 – Supplemental material for Digital dementia training for formal caregivers: a scoping review of platforms, outcomes and evidence gaps Supplemental material, sj-xlsx-1-jrn-10.1177_17449871261495844 for Digital dementia training for formal caregivers: a scoping review of platforms, outcomes and evidence gaps by Clare Barrett, Wendy Moyle, Jonathan Foo, Marie-Claire O’Shea and Lana Mitchell in Journal of Research in Nursing

sj-xlsx-2-jrn-10.1177_17449871261495844 – Supplemental material for Digital dementia training for formal caregivers: a scoping review of platforms, outcomes and evidence gaps Supplemental material, sj-xlsx-2-jrn-10.1177_17449871261495844 for Digital dementia training for formal caregivers: a scoping review of platforms, outcomes and evidence gaps by Clare Barrett, Wendy Moyle, Jonathan Foo, Marie-Claire O’Shea and Lana Mitchell in Journal of Research in Nursing
