# Supplementary material for: Digital dementia training for formal caregivers: a scoping review of platforms, outcomes and evidence gaps
Source: J Res Nurs. 2026 Nov 7:17449871261495844. Online ahead of print. doi: 10.1177/17449871261495844 (PMC13744493; doi:10.1177/17449871261495844)
Supplement: Supplementary file 1 [file sj-docx-1-jrn-10.1177_17449871261495844.docx]

**Supplementary File 1 – Search Translation Summary**

# cinahl complete (ebsco)

### Search Strategy:

| **# 13** | **414** | **#3 and #6 and #9 and #12** |
| --- | --- | --- |
| # 12 | 1,213,851 | #10 or #11 |
| # 11 | 141,577 | MH ((caregivers OR health personnel OR occupational groups OR caregiver OR nurses OR personnel OR worker OR Nurses' Aides – psychology OR workforce OR care giver OR care aide OR care assist* ) OR care employee OR nurses aide OR nursing assistant OR personal care worker* OR PCW OR AIN OR assistant in nursing) |
| # 10 | 1,166,253 | XB (caregiver* OR Carer* OR nurs* OR worker* OR staff OR assist* OR personnel OR aide OR employee OR PCW OR AIN OR support OR attendant OR RN Or EN OR orderly) |
| # 9 | 179,330 | #7 or #8 |
| # 8 | 46,261 | MH (education, distance OR Internet -- organization & administration OR online instruction OR application OR distance learning OR internet OR distance education OR online learning OR learning online OR online education OR education online) |
| # 7 | 151,601 | XB (online OR digital OR (microlearning or micro-learning or micro learning or microeducation) OR bite-sized learning OR (spaced learning or spaced education or spaced repetition) OR distributed learning OR (just-in-time learning or JIT learning) OR nano learning OR microcontent OR micro-content OR microcourse OR “micro course” OR learning nuggets OR mobile microlearning OR app-based learning) |
| # 6 | 499,778 | #4 or #5 |
| # 5 | 9,519 | MH (education OR inservice training OR training OR continuing education OR clinical competence education OR Continuing -- organization & administration OR geriatric nursing education OR Nurses' Aides -- education OR job training OR educational programs OR competency based learning) |
| # 4 | 495,003 | XB (training OR education) |
| # 3 | 104,009 | #1 or #2 |
| # 2 | 71,656 | MH (dementia OR dementia nursing OR alzheimer's disease OR Neurocognitive Disorders OR Geriatric Psychiatry) |
| # 1 | 90,249 | XB (Dementia  OR Alzheimer* OR BPSD OR “behavioural and psychological symptoms of dementia”) |

Limits: Peer reviewed, English Language

# medline (ebsco)

### Search Strategy:

| **# 13** | **716** | **#3 and #6 and #9 and #12** |
| --- | --- | --- |
| # 12 | 3,267,533 | #10 or #11 |
| # 11 | 202,037 | MH ((caregivers OR health personnel OR occupational groups OR caregiver OR nurses OR personnel OR worker OR Nurses' Aides – psychology OR workforce OR care giver OR care aide OR care assist* ) OR care employee OR nurses aide OR nursing assistant OR personal care worker* OR PCW OR AIN OR assistant in nursing) |
| # 10 | 3,200,943 | XB (caregiver* OR Carer* OR nurs* OR worker* OR staff OR assist* OR personnel OR aide OR employee OR PCW OR AIN OR support OR attendant OR RN Or EN OR orderly) |
| # 9 | 544,219 | #7 or #8 |
| # 8 | 80,349 | MH (education, distance OR Internet -- organization & administration OR online instruction OR application OR distance learning OR internet OR distance education OR online learning OR learning online OR online education OR education online) |
| # 7 | 491,399 | XB (online OR digital OR (microlearning or micro-learning or micro learning or microeducation) OR bite-sized learning OR (spaced learning or spaced education or spaced repetition) OR distributed learning OR (just-in-time learning or JIT learning) OR nano learning OR microcontent OR micro-content OR microcourse OR “micro course” OR learning nuggets OR mobile microlearning OR app-based learning) |
| # 6 | 1,174,868 | #4 or #5 |
| # 5 | 36,476 | MH (education OR inservice training OR training OR continuing education OR clinical competence education OR Continuing -- organization & administration OR geriatric nursing education OR Nurses' Aides -- education OR job training OR educational programs OR competency based learning) |
| # 4 | 1,156,179 | XB (training OR education) |
| # 3 | 307,754 | #1 or #2 |
| # 2 | 63,438 | MH (dementia OR dementia nursing OR alzheimer's disease OR Neurocognitive Disorders OR Geriatric Psychiatry) |
| # 1 | 293,974 | XB (Dementia  OR Alzheimer* OR BPSD OR “behavioural and psychological symptoms of dementia”) |

# PROQUEST CENTRAL (PROQUEST)

### Search Strategy: ((((((title(Dementia  OR Alzheimer* OR BPSD OR "behavioural and psychological symptoms of dementia") OR abstract(Dementia  OR Alzheimer* OR BPSD OR "behavioural and psychological symptoms of dementia")) AND la.exact("English")) AND peer(yes)) AND la.exact("English")) AND PEER(yes)) OR (mainsubject(dementia OR dementia nursing OR alzheimer's disease OR Neurocognitive Disorders OR Geriatric Psychiatry) AND PEER(yes) AND la.exact("English"))) AND ((((((abstract(training OR education) OR title(training OR education)) AND la.exact("English")) AND peer(yes)) AND la.exact("English")) AND PEER(yes)) OR (mainsubject(education OR inservice training OR training OR continuing education OR clinical competence education OR Continuing organization administration OR geriatric nursing education OR Nurses' Aides education OR job training OR educational programs OR competency based learning) AND PEER(yes) AND la.exact("English"))) AND ((((((abstract(online OR digital OR (microlearning OR micro-learning OR micro learning OR microeducation) OR bite-sized learning OR (spaced learning OR spaced education OR spaced repetition) OR distributed learning OR (just-in-time learning OR JIT learning) OR nano learning OR microcontent OR micro-content OR microcourse OR "micro course" OR learning nuggets OR mobile microlearning OR app-based learning) OR title(online OR digital OR (microlearning OR micro-learning OR micro learning OR microeducation) OR bite-sized learning OR (spaced learning OR spaced education OR spaced repetition) OR distributed learning OR (just-in-time learning OR JIT learning) OR nano learning OR microcontent OR micro-content OR microcourse OR "micro course" OR learning nuggets OR mobile microlearning OR app-based learning)) AND la.exact("English")) AND peer(yes)) AND la.exact("English")) AND PEER(yes)) OR (mainsubject(education, distance OR Internet organization administration OR online instruction OR application OR distance learning OR internet OR distance education OR online learning OR learning online OR online education OR education online) AND PEER(yes) AND la.exact("English"))) AND ((((((abstract(caregiver* OR Carer* OR nurs* OR worker* OR staff OR assist* OR personnel OR aide OR employee OR PCW OR AIN OR support OR attendant OR RN OR EN OR orderly) OR title(caregiver* OR Carer* OR nurs* OR worker* OR staff OR assist* OR personnel OR aide OR employee OR PCW OR AIN OR support OR attendant OR RN OR EN OR orderly)) AND la.exact("English")) AND peer(yes)) AND la.exact("English")) AND PEER(yes)) OR (mainsubject((caregivers OR health personnel OR occupational groups OR caregiver OR nurses OR personnel OR worker OR Nurses' Aides psychology OR workforce OR care giver OR care aide OR care assist*) OR care employee OR nurses aide OR nursing assistant OR personal care worker* OR PCW OR AIN OR assistant in nursing) AND PEER(yes) AND la.exact("English")))

Results 1,002

# SCOPUS

### Search Strategy:

TITLE-ABS-KEY((dementia OR Alzheimer* OR BPSD OR "behavioural and psychological symptoms of dementia" OR "dementia nursing" OR "alzheimer's disease" OR "neurocognitive disorders" OR "geriatric psychiatry") AND (training OR education OR "inservice training" OR "continuing education" OR "clinical competence education" OR "geriatric nursing education" OR "job training" OR "educational programs" OR "competency based learning") AND (online OR digital OR microlearning OR "micro-learning" OR "micro learning" OR microeducation OR "bite-sized learning" OR "spaced learning" OR "spaced education" OR "spaced repetition" OR "distributed learning" OR "just-in-time learning" OR "JIT learning" OR nano learning OR microcontent OR "micro-content" OR microcourse OR "micro course" OR "learning nuggets" OR "mobile microlearning" OR "app-based learning" OR "distance education" OR "online instruction" OR "distance learning" OR "online learning" OR "online education") AND (caregiver* OR carer* OR nurs* OR worker* OR staff OR assist* OR personnel OR aide OR employee OR PCW OR AIN OR support OR attendant OR RN OR EN OR orderly OR "health personnel" OR "nurses' aides" OR "nursing assistant" OR "personal care worker*" OR "assistant in nursing")) AND (LIMIT-TO(LANGUAGE, "English")) AND (LIMIT-TO(DOCTYPE, "ar"))

Results 227
